# Differential local tissue permissiveness influences the final fate of GPR17‐expressing oligodendrocyte precursors in two distinct models of demyelination

**DOI:** 10.1002/glia.23305

**Published:** 2018-02-09

**Authors:** Giusy T. Coppolino, Davide Marangon, Camilla Negri, Gianluca Menichetti, Marta Fumagalli, Paolo Gelosa, Leda Dimou, Roberto Furlan, Davide Lecca, Maria P. Abbracchio

**Affiliations:** ^1^ Laboratory of Molecular and Cellular Pharmacology of the Purinergic Transmission, Dipartimento di Scienze Farmacologiche e Biomolecolari Università degli Studi di Milano, Via Balzaretti 9 Milan 20133 Italy; ^2^ Centro Cardiologico Monzino, Via Parea, 4 Milano 20138 Italy; ^3^ Molecular and Translational Neuroscience University of Ulm, Albert‐Einstein‐Allee 11 Ulm D – 89081 Germany; ^4^ Institute of Experimental Neurology, S. Raffaele Scientific Institute, Via Olgettina, 58 Milano 20132 Italy

**Keywords:** animal models, differentiation, G protein‐coupled receptor, multiple sclerosis, oligodendrocyte precursor cells

## Abstract

Promoting remyelination is recognized as a novel strategy to foster repair in neurodegenerative demyelinating diseases, such as multiple sclerosis. In this respect, the receptor GPR17, recently emerged as a new target for remyelination, is expressed by early oligodendrocyte precursors (OPCs) and after a certain differentiation stage it has to be downregulated to allow progression to mature myelinating oligodendrocytes. Here, we took advantage of the first inducible GPR17 reporter mouse line (GPR17‐iCreER^T2^xCAG‐eGFP mice) allowing to follow the final fate of GPR17^+^ cells by tamoxifen‐induced GFP‐labeling to unveil the destiny of these cells in two demyelination models: experimental autoimmune encephalomyelitis (EAE), characterized by marked immune cell activation and inflammation, and cuprizone induced demyelination, where myelin dysfunction is achieved by a toxic insult. In both models, demyelination induced a strong increase of fluorescent GFP^+^ cells at damaged areas. However, only in the cuprizone model reacting GFP^+^ cells terminally differentiated to mature oligodendrocytes, thus contributing to remyelination. In EAE, GFP^+^ cells were blocked at immature stages and never became myelinating oligodendrocytes. We suggest these strikingly distinct fates be due to different permissiveness of the local CNS environment. Based on previously reported GPR17 activation by emergency signals (e.g., Stromal Derived Factor‐1), we propose that a marked inflammatory milieu, such as that reproduced in EAE, induces GPR17 overactivation resulting in impaired downregulation, untimely and prolonged permanence in OPCs, leading, in turn, to differentiation blockade. Combined treatments with remyelinating agents and anti‐inflammatory drugs may represent new potential adequate strategies to halt neurodegeneration and foster recovery.

## 1 INTRODUCTION

Oligodendroglial precursor cells (OPCs), also known as NG2 cells due to their expression of the proteoglycan NG2, are known to proliferate and participate to remyelination during multiple sclerosis. However, while disease progresses, this process becomes progressively less efficient, eventually resulting in blockade of oligodendroglial differentiation and impaired myelin repair (Podbielska, Banik, Kurowska, & Hogan, 2013). This, in turn, leads not only to overt neurological disturbances due to interruption of impulse transmission, but also to axonal damage and neurodegeneration followed by progression of the remitting‐relapsing to the progressive chronic form of the disease. The reasons responsible for the progressive loss of patients’ remyelinating abilities are still under study. Thus promoting myelination via a specific action on key molecules involved in OPC maturation has been increasingly recognized as a promising strategy to foster endogenous remyelination, especially in the progressive forms (Rovaris et al., 2006).

In this respect, the G protein‐coupled receptor GPR17 has recently emerged as a key timer of oligodendrogliogenesis. In the intact brain and spinal cord, GPR17 is specifically expressed by a subset of early bipolar NG2 positive (GPR17^+^‐NG2^+^) OPCs, accompanies OPC maturation up to immature/pre‐oligodendrocytes and is then downregulated before terminal maturation (Lecca et al., 2008; Ceruti et al., 2009). Any alterations in this precise expression pattern result in myelination defects (Chen et al., 2009; Fumagalli et al., 2011; Fumagalli et al., 2015). Pathologically increased levels of GPR17 have been found after focal lysolecithin induced demyelination (Boda et al., 2011), following brain ischemia in rodents subjected to middle cerebral artery occlusion (MCAO) (Ciana et al., 2006; Lecca et al., 2008) and after traumatic brain injury in both rodents (Boda et al., 2011) and human subjects (Franke et al., 2013). In initial studies, the mRNA for GPR17 was also found to be increased in the spinal cord of mice with experimental autoimmune encephalomyelitis (EAE) and in the spinal lesions of multiple sclerosis patients (Chen et al., 2009). The exact significance of persistent GPR17 overexpression *in vivo* under demyelinating conditions is still unknown. Our *in vitro* data suggest that GPR17 aberrant overexpression in OPCs leads to impaired downregulation at late differentiation stages and blockade of cells at immature stages, thus impairing remyelination (Fumagalli, Lecca, & Abbracchio, 2016). Initial data also suggest that GPR17 overexpression may be due, at least in part, to inflammatory cytokines and chemokines like the stromal derived factor 1 (SDF1), that accumulate at the sites of demyelinating inflamed lesions (Calderon et al., 2006) and can indeed specifically interact with this receptor (Parravicini et al., 2016). However, a detailed study on GPR17 dysfunction in *in vivo* conditions associated to demyelination and inflammation is still missing, nor is known whether changes in the pool of GPR17^+^ cells contribute to, or contrast, lesion repair.

Here, to shed light on this issue, we used two rodent demyelination models: the autoimmune experimental encephalomyelitis (EAE) model, the most studied animal model of human multiple sclerosis over the past decades, and the cuprizone‐induced model, that bypasses the autoimmune component and is more adequate to study the timing of demyelination and remyelination phases (Denic et al., 2011). To detail the final fate of the GPR17‐expressing cells and to unveil whether they indeed become functionally mature myelinating oligodendrocytes, we took advantage of the first inducible fluorescent GPR17 reporter mouse line for fate mapping studies (GPR17‐iCreER^T2^xCAG‐eGFP mice), where, upon tamoxifen treatment, all GPR17‐expressing cells at that specific moment become permanently fluorescent, and can be traced throughout life (Viganò et al., 2016). Since, as already mentioned, GPR17 is only transiently expressed by OPCs and is no longer present in mature myelinating oligodendrocytes (Lecca et al., 2008; Fumagalli et al., 2011; Fumagalli et al., 2015) this transgenic mouse line represents the only possible means to follow *in vivo* the final destiny of the GPR17^+^ pool of OPCs, even after cells have physiologically downregulated the receptor along their differentiation pathway.

We show that, in both models of demyelination, the pool of GPR17^+^ OPCs rapidly responds by increasing the number of GFP^+^ cells accumulating at demyelinated lesions, but that only in the cuprizone model these activated cells can successfully proceed to terminal differentiation and start expressing myelin proteins. Instead, in EAE, GFP^+^ cells are blocked at immature stages and do not express mature myelin markers. We postulate that the inflammatory environment could be responsible for the lack of GPR17 down‐regulation and the subsequent remyelination failure.

## 2 MATERIALS AND METHODS

### 2.1 Animal care

For all animal studies, international (European law Dir. 2010/63/UE) and national (Italian law DL n. 26, 4th March 2014) guidelines for the care and use of animals were followed. All the procedures were approved by the Italian Ministry of Health (authorization 473–2015PR to MPA).

Mice were housed in groups of 4, under a 12‐hr light/12‐hr dark cycle at 21°C, with food and water *ad libitum*. In selected experiments we used wild‐type mice (purchased from Charles River, Calco, Italy) or adult animals from the GPR17‐iCreER^T2^xCAG‐eGFP mouse line. In this case mice were maintained and bred at our local facilities. Offspring were ear punched and genotyped using PCR as previously reported (Viganò et al., 2016). The body weight of the mice was monitored during all experiments.

### 2.2 Experimental design

The experiments were designed in compliance with the ARRIVE guidelines. Control groups were included in all experiments, randomizing the procedures and applying double‐blinded analysis when possible. Sample size was calculated with G‐Power, considering a significant level of .05. Based on our preliminary results, to reach a power between 0.80 and 0.90, we needed a minimum of *n* = 4 (immunohistochemistry, RT‐PCR), depending on the specific experimental conditions. This number is in line to those generally employed in the field.

### 2.3 EAE induction

EAE was induced in 8‐week‐old female wild‐type C57Bl/6 mice (Charles River) by subcutaneous immunization in the flanks and in the tail base with 200 μg of myelin oligodendrocyte glycoprotein (MOG35–55, Espikem, Florence, Italy) per mouse in incomplete Freund's adjuvant (IFA, Sigma‐Aldrich, Milan, Italy) supplemented with 8 mg/ml of Mycobacterium tuberculosis (strain H37Ra, BD Difco, Milan, Italy). The immunized mice received intravenously 500 ng of pertussis toxin (PTX, Duotech, Milan, Italy) the day of the immunization and 48 hr later. Animals were daily weighted and scored for clinical symptoms of EAE according the following scale (clinical score, CS): 0 = healthy, 1 = flaccid tail, 2 = ataxia and/or paresis of hindlimbs, 3 = paralysis of hindlimbs and/or paresis of forelimbs, 4 = tetraparalysis, 5 = moribund or death. Non‐EAE age‐matched controls received PTX injections, as well as the initial injections of emulsion but without the encephalitogen, to ensure that observed effects are due to EAE and not to a non‐specific reaction to the ancillary components that are used to facilitate disease induction. The animals were sacrificed 21 days post immunization (dpi) for histological and real‐time PCR analysis. For the analysis only animals with CS > 1.5 were considered. The use of female mice was based on the documented higher response to MOG immunization. Moreover, higher aggressiveness of C57BL/6 male mice was reported to increase stress, with production of corticosteroids that can inhibit EAE development (Miller & Karpus 2007). Also for this reason, the use of female mice is usually preferred.

### 2.4 Cuprizone‐induced demyelination

Eight‐week‐old age male C57BL/6 wild‐type mice (Charles River) were fed with 0.2% (weight/weight) cuprizone‐supplemented diet (Sigma‐Aldrich) *ad libitum* for 5 weeks and were then switched to normal diet for further 3 weeks to allow spontaneous remyelination (Gudi, Gingele, Skripuletz, & Stangel, 2014). Immunohistochemistry and qRT‐PCR analysis were performed at the beginning of the treatment (W0) and after 1, 3, 5, and 7 weeks. Although the course of de‐ and remyelination is similar between genders in C57BL/6 mice, the use of male mice is more well‐documented in the literature (Taylor, Gilmore, Ting, & Matsushima, 2010).

### 2.5 Tamoxifen induction

Tamoxifen (40 mg/ml; Sigma‐Aldrich) was diluted in ethanol (final concentration 10%) and corn oil. Every other day mice received for three times 10 mg of tamoxifen suspension by oral gavaging (for a total of 30 mg) 2 weeks before EAE induction and were sacrificed 21 dpi for histological and real‐time PCR analysis. In case of cuprizone‐induced demyelination, GFP expression with tamoxifen was induced at week 3, as described above.

### 2.6 Histological analysis, immunofluorescence and cell counts

Mice were anesthetized with ketamine (100 mg/kg)/xylazine (10 mg/kg) and perfused transcardially with saline‐0,1 M EDTA (Sigma Aldrich) followed by 4% neutral buffered formalin (Sigma Aldrich) in deionized water. Spinal cords were collected and post‐fixed for 1 hr in the same solution at 4°C, cryoprotected in 30% sucrose for 24 hr (until the tissue sinks to the bottom of the tube), embedded in OCT and then frozen at −80°C. Spinal cords were cut transversally into 20 µm‐thick sections with a cryostat and processed for immunofluorescence.

Slides were incubated for 45 min at room temperature with a blocking solution composed by 10% normal goat serum and 0.1% triton X‐100 in 1× phosphate buffered saline (PBS).

Then, the sections were incubated with primary antibodies overnight at 4°C in 1× PBS with 5% goat normal serum and 0.1% Triton X‐100. The following primary antibodies were used: rabbit polyclonal anti‐GPR17 (1:2,500, custom antibody produced by PRIMM, Milan, Italy), anti‐NG2 (1:2,000, Millipore, Milan, Italy), anti‐OLIG2 (1:500, Millipore), anti‐IBA1 (1:500; Wako, Japan), anti‐GSTπ (1:300, MBL, Woburn, USA), chicken polyclonal anti‐GFP (1:1,400, Aves Labs, Tigard, Oregon, USA), and mouse monoclonal anti‐GFAP (1:500, Cell Signalling, Danvers, USA), anti‐CC1 (1:50, Millipore), anti‐MAP2 (1:500, Immunological sciences, Rome, Italy), anti‐NEUN (1:100, Millipore).

Following primary antibody incubation, the sections were washed and incubated with the appropriate biotinylated secondary antibody (Vector Labs, Burlingame, USA), and/or with Alexa 405, 488, or 568 conjugated secondary antibody or streptavidin (Invitrogen, Molecular Probes, Monza, Italy) for 1 hr at room temperature. Hoechst 33528 was used to visualize cell nuclei. After processing, sections were mounted on microscope slides with fluorescent mounting medium (Dako, Milan, Italy). GPR17 labeling was amplified with the high sensitivity tyramide signal amplification kit (Perkin Elmer, Milan, Italy) according to the manufacturer's instruction.

After immunofluorescence, one section from each level (cervical, thoracic, and lumbar‐ sacral) of the spinal cord was analyzed for each animal. Seven arbitrary non‐overlapping sample fields were counted separately in white matter (WM) and six fields in gray matter (GM) of every section at 40× magnification.

For the correlation analysis GFP^+^ cells were counted in 41 fasciculus gracilis area of EAE mice in whole spinal cord (cervical, thoracic, and lumbar‐sacral levels). Fluoromyelin red stain (1:300 in 1× PBS; Thermofisher Scientific, Milan, Italy) was used to selectively label myelin in this area.

Luxol fast blue staining was performed to mark myelin in the corpus callosum of mice (Supporting Information Figure S1). In detail, sections were acclimatized at room temperature for 20 min and then dehydrated by passing the slides through growing graded alcoholic solutions (70, 90, 100% EtOH), 2 min each. Slides were then incubated with Luxol Fast Blue solution (Sigma Aldrich; 1 mg/ml in 95% EtOH, 0.5% acetic acid) over‐night at 56°C. The next day, the excess stain was rinsed off first with 95% EtOH and then with distilled water, 30 s each. The differentiation step was performed first in lithium carbonate solution and then in 70% EtOH, 30 s each, and finally slides were rinsed in distilled water. Differentiation step was repeated till GM was clear and WM sharply defined by the blue stain in control condition. Hematoxylin (Sigma Aldrich) was used to counterstain sections. Slides were mounted with DPX mountant for histology (Sigma Aldrich).

### 2.7 *In situ* hybridization

Slides were dried at room temperature for 5 min. After one wash in 1X PBS‐DEPC, slides were post‐fixed in 4% paraformaldehyde for 5 min, washed in 1× PBS‐DEPC, washed for 10 min with 2× SSC and then incubated in Tris‐Glycine 0.1M (pH 7) for 20 min. Sections were permeabilized in protease K (20 μg/ml in 1× PBS) for 10 min at 37°C. The activity of protease K was stopped by fixation in 4% paraformaldehyde for 5 min, followed by two 5‐min washes in 1× PBS. Slides were incubated in 0.25% acetic diaminobenanhydride (Sigma‐Aldrich) with 0.1M triethanoloamine (pH 8.0; Sigma‐Aldrich) for 10 min at room temperture, and then washed in 2× SSC for 10 min. Digoxigenin‐labeled cRNA (0.3 μg/ml) of either antisense or sense probes were added to hybridization buffer containing 50% formamide, 10% dextran sulphate, 1× Denhardt's solution, 4× SSC, 250 μg/ml salmon sperm, 10 mM DTT and 250 μg/ml *Escherichia coli* tRNA. Hybridization was carried out for 16 hr at 55°C in hybridization oven. The sections were incubated in 2× SSC for 30 min at 52°C, quickly washed twice in 0.2× SSC, and then cooled in 1× PBS at room temperature. The following protocol was used to detect the hybridization signals. First, the sections were incubated in blocking buffer containing 3% bovine serum albumin (Sigma‐Aldrich) and 0.1% Triton X‐100 in PBS at room temperature for 30 min, and then with anti‐digoxigenin sheep IgG Fab fragments conjugated to alkaline phosphatase (Roche, Monza, Italy) diluted 1:500 in the blocking buffer o/n. Slides were washed 3 times with 1× PBS, then in Buffer 1 (0.1M Tris‐HCl, pH 7.5, 0.1M NaCl) and finally equilibrated in Buffer 2 (0.1M Tris‐HCl, pH 9.5, 0.1M NaCl, 0.05M MgCl). Color development was obtained with 400 μg/ml nitro blue tetrazolium, 200 μg/ml 5‐bromo‐4‐chloro‐3‐indolyl phosphate and 100 μg/ml levamisole in buffer 2 in the dark at room temperture for 10 min. The sections were rinsed in distilled water to stop color development, and then dried by soaking the slides through growing graded alcoholic solutions (50%, 75%, 95%, and 100%) and xylene for 10 min. Slides were mounted with DPX (Sigma‐Aldrich).

Mouse GPR17 cDNA sequences (sense: 5′ GATGAACGGTCTGGAGGCAGCC 3′; antisense: 5′ CTCACAGCTCGGATCGGGCAC 3′) were inserted in a pBlu2KSM‐T vector (Clontech, USA). Digoxigenin‐labeled RNA probes were synthesized following the manufacturer's instructions (Roche, Monza, Italy).

### 2.8 Total RNA extraction, retrotranscription and gene expression analysis

Total RNA was extracted from cells or tissues using Trizol reagent (Life Technologies, Monza, Italy). For gene expression analysis, cDNA synthesis was performed starting from 800 ng of total RNA using SuperScript II Reverse Transcriptase (Life Technologies). The expression of all genes was analysed using Sybr‐green reagents (Bio‐rad, Milan, Italy) and normalized to GAPDH expression using CFX96 real‐time PCR system (Bio‐rad) following the manufacturer's protocol. The Ct values were elaborated with the comparative CT method (ΔΔCT) which allows the relative quantification of template comparing the expression levels of the interested gene with the ones of the housekeeping gene.

### 2.9 Statistical analysis

Data are presented as mean ± *SEM* and analyzed with the GraphPad Prism 6.0 software. For all comparisons between two groups with a normal distribution, two‐tailed unpaired Student *t*‐test was performed. For multiple comparison testing, one‐way analysis of variance (ANOVA) accompanied by Tukey's post‐hoc test was used. Differences were considered significant for *p* < .05. Correlation analysis between the number of GFP^+^ cells and the extent of demyelination in the fasciculus gracilis area was performed by applying the Pearson's correlation test.

## 3 RESULTS

### 3.1 In mouse spinal cord, GPR17 is expressed in cells of the oligodendroglial lineage

**Figure 1:**
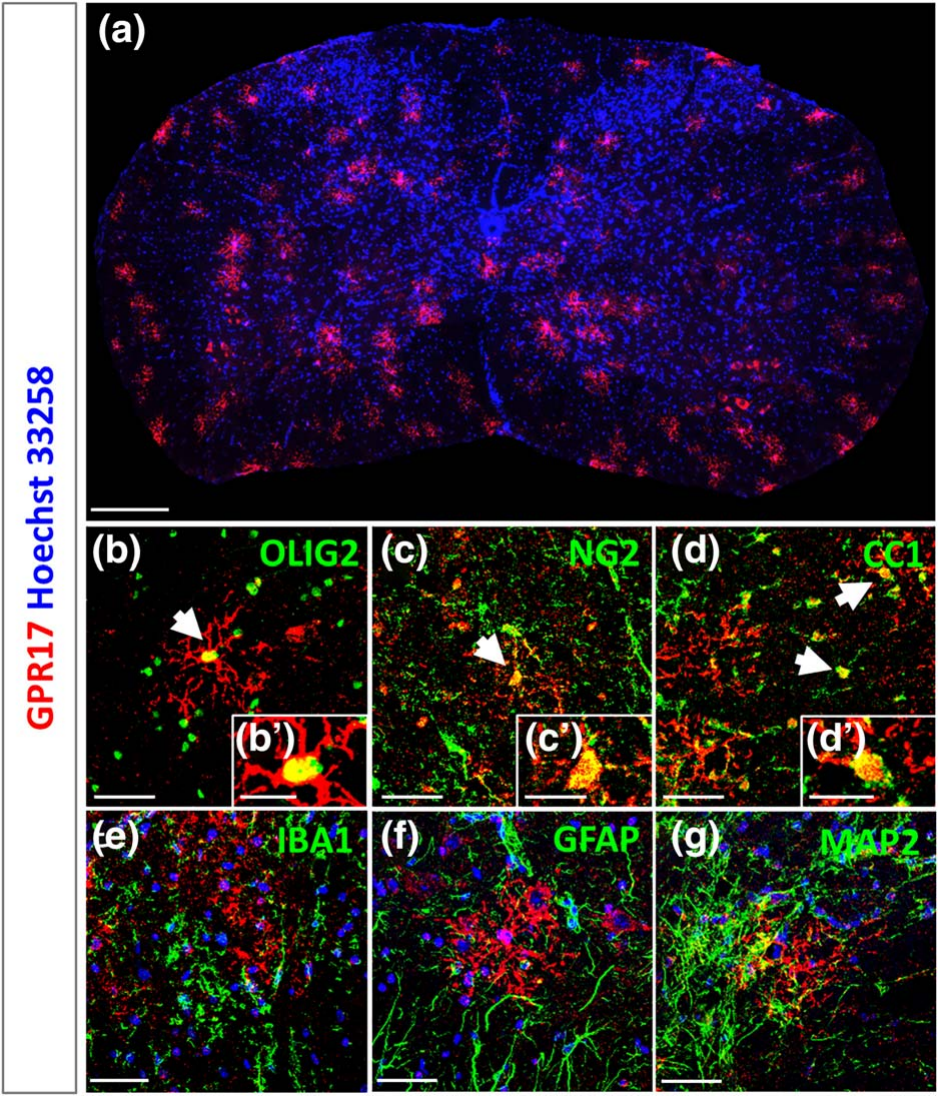
Confocal images of control mouse spinal cord immunolabeled for GPR17 and other markers. (a) Distribution of GPR17^+^‐cells (in red) in the spinal cord of an adult mouse. Cell nuclei were labeled with Hoechst 33258 (in blue). GPR17^+^‐cells expressed the oligodendroglial marker OLIG2 (b and inset b′). GPR17 staining showed co‐localization with both the early OPC marker NG2 (c and inset c′) and the more mature marker CC1 (d and inset d′). No GPR17 positivity was found in microglia (IBA1^+^ cells), astrocytes (GFAP^+^ cells) and neurons (MAP2^+^ cells) (e, f, and g). Micrographs were taken at the confocal microscope (Zeiss LSM 510 Meta). Scale bars: 200 µm (a), 50 µm (b–g), 20 µm (b′, c′, and d′) [Color figure can be viewed at http://wileyonlinelibrary.com]

As a first step, we characterized the expression pattern of GPR17 in spinal cord of adult mice. The GPR17 receptor protein was present in a relatively high number of cells throughout the whole spinal cord (Figure 1a), where it clearly decorated oligodendroglial cells, as confirmed by co‐localization with the typical oligodendroglial transcription factor OLIG2 (Figure 1b,b′). In particular, GPR17 is expressed in early OPCs with a bipolar phenotype positive for NG2, but also in maturing oligodendrocyte precursors positive for CC1 (Figure 1c,c′,d,d′). No co‐localization was found with either microglial, astrocytic or neuronal markers, like IBA1, GFAP, and MAP2, respectively (Figure 1e–g). Thus, we confirmed that also in spinal cord, as previously shown for brain (Lecca et al., 2008; Boda et al., 2011; Ceruti et al., 2011), GPR17 is specifically expressed in a subset of oligodendrocyte precursors at intermediate stages of differentiation.

### 3.2 GPR17‐expressing OPCs respond to EAE induction but do not reach terminal maturation

**Figure 2:**
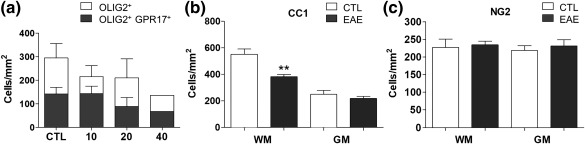
Oligodendrocyte alterations during EAE. (a) Animals were analyzed on 10, 20, and 40‐day post immunization (DPI). We reported oligodendrocyte (OLIG2^+^‐cells) loss at each time‐point as expected in this disease; most of these cells were GPR17‐expressing OPCs and immature oligodendrocytes; in particular, upon acute EAE (20 DPI) there was a significant decrease of CC1^+^ mature oligodendrocytes (b). At this time, the NG2^+^ OPCs were almost the same in both white (WM) and gray (GM) matter (c). Unpaired two‐tailed Student's *t* test; **p < .01 compared with control from two independent experiments

To unveil disease related changes of GPR17 mRNA and protein, we induced EAE in mice and followed disease development at 10, 20, and 40 days post immunization (DPI). As expected, during EAE development, we observed a progressive loss of cells belonging to the oligodendrocyte lineage. This initial general analysis showed a trend to decrease in the number of OLIG2^+^ cells, many of which were also positive for GPR17 that indeed decorated heterogeneous subsets of immature oligodendrocytes (Figure 2a). For our subsequent studies, we focused on the acute phase, when spinal cord inflammation is high, clinical symptoms severe and the highest decrease in OLIG2 is accompanied by a strong reduction in the number of the GPR17^+^ cell pool. Mice were sacrificed 20 days after EAE induction, spinal cords were explanted and analyzed by both qRT‐PCR and immunohistochemistry in both WM and GM.

**Figure 3:**
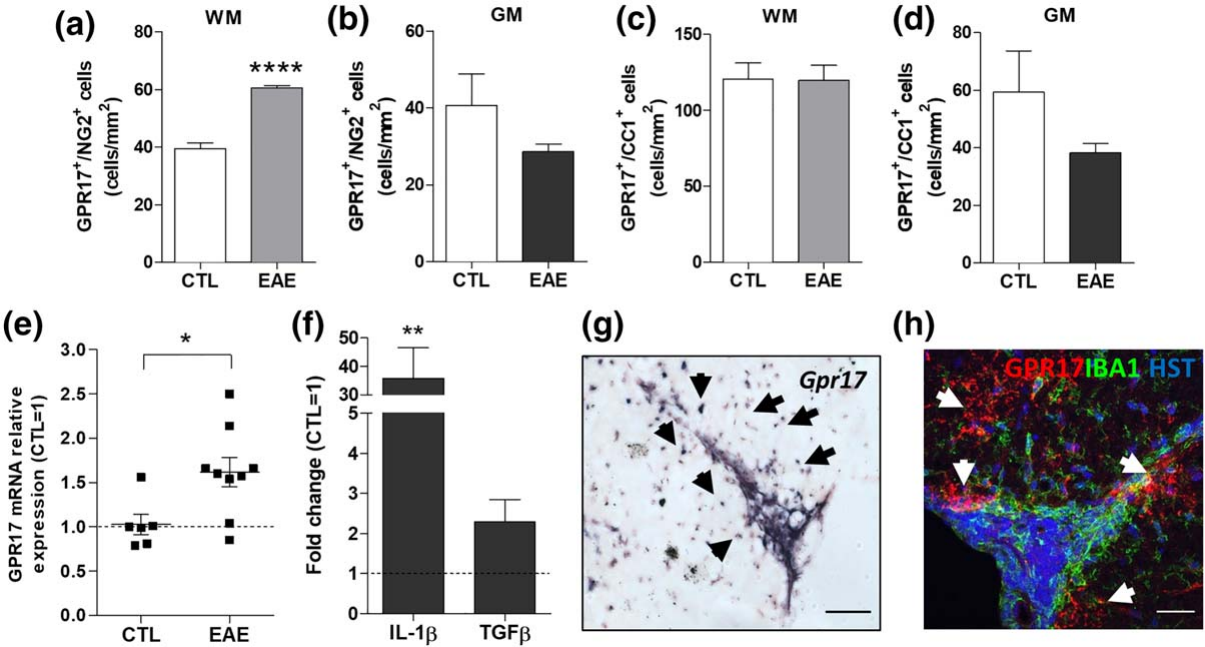
Characterization of GPR17 subpopulations and changes of GPR17 expression in acute EAE in spinal cord. Animals were analyzed 21 days after immunization (acute EAE). Upon acute phase, there was a significant increase of GPR17^+^/NG2^+^ cells (OPCs), only in the white matter (WM; a and b); conversely, the number of GPR17^+^/CC1^+^ cells (immature oligodendrocytes) was apparently unchanged in both WM and GM (c, d). Data are the mean ± *SEM* of cervical, thoracic and lumbar sections; (CTL *n* = 3, EAE *n* = 5). Unpaired two‐tailed Student's *t* test; ****p < .0001 compared with control from two independent experiments. (e) By means of real‐time PCR, a significant up‐regulation of GPR17 was found in spinal cord of mice after acute EAE compared with controls and this correlated with the increased expression of inflammatory cytokines (f). Histograms show the fold change value ± *SEM* compared with control set to 1. Two‐tailed Mann‐Whitney student *t* test, *p ≤ .05, **p < .01 from three independent experiments. (g) GPR17 up‐regulation was also confirmed by means of *in situ* hybridization at the lesion site (black arrows) indicate cells with increased levels of *Gpr17* mRNA. Scale bar 100 μm. (h) A local up‐regulation of GPR17^+^ cells (white arrows) was observed after EAE induction in the same area where inflammatory cells infiltrate the tissue (characterized by a high number of nuclei, in blue), bordered by IBA1^+^ activated microglial cells. Scale bar 50 μm [Color figure can be viewed at http://wileyonlinelibrary.com]

At this time point, we observed a significant decrease of CC1^+^‐mature oligodendrocytes (Figure 2b) in the WM (but not in the GM), whereas the number of early NG2^+^ OPCs was almost unaltered (Figure 2c). However, the subset of the GPR17^+^/NG2^+^ cells (i.e., intermediate precursors), was markedly increased, indicating that these cells specifically respond to injury. Of note, this increase was evident only in the WM, but not in the GM (Figure 3a,b) that is relatively spared in this model, confirming this behavior be restricted to the regions where demyelination occurs. Despite this increase, the number of more advanced GPR17^+^/CC1^+^ cells was apparently unchanged in both WM and GM (Figure 3c,d) suggesting that these cells cannot proceed any further.

Real‐time PCR analysis showed that *Gpr17* mRNA was almost doubled in EAE spinal cord compared with control (Figure 3e), along with two inflammatory cytokines (i.e., *Il‐1β* and *Tgf‐β*), which confirmed the presence of strong acute inflammation (Figure 3f). Increased *Gpr17* gene expression was also confirmed by *in situ* hybridization analysis showing *Gpr17*‐expressing cells accumulating at the borders of EAE infiltration sites (Figure 3g). Immunofluorescence analysis confirmed that increased levels of *Gpr17* mRNAs are followed by increased receptor protein synthesis, as shown by the large presence of cells expressing the GPR17 protein at demyelinating foci, where inflammatory cells expressing the typical microglia/monocyte marker IBA1 were also found (Figure 3h). Globally, these data suggest that, (i) as expected, EAE induction greatly affects oligodendrocytes, and that (ii) the subset of GPR17^+^ OPCs rapidly reacts to the demyelinating insult, likely to replace dying cells.

**Figure 4:**
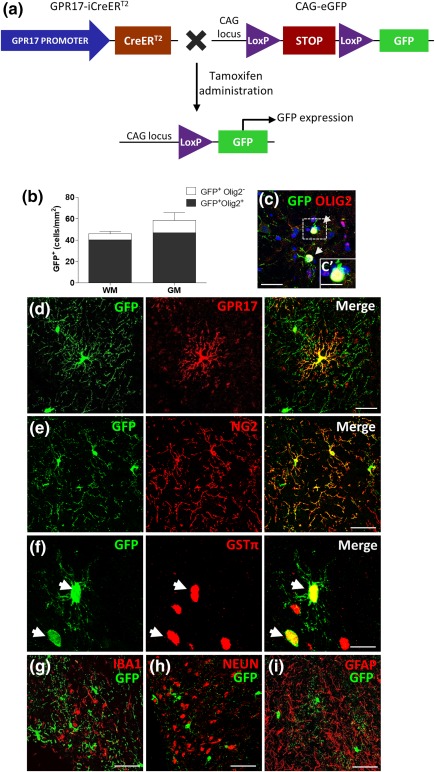
Identity of recombinant cells in the adult spinal cord. (a) Schematic representation of the transgenic alleles in GPR17‐iCreER^T2^xCAG‐eGFP mice showing tamoxifen‐responsive recombination of the CAG‐GFP allele to induce GFP in cells expressing GPR17. (b and c) Quantification of OLIG2^+^ cells among GFP^+^ cells in the spinal cord reveals that nearly all recombined cells belong to the oligodendroglial lineage. Many GFP^+^ cells were also found positive for GPR17 (d) and the NG2 marker (e). Although they were not abundant, we detect the presence of some GFP^+^GST^+^ cells (f). Vice versa, GFP^+^ cells are not positive for the markers of microglia, IBA1 (g), neurons, NEUN (h) and astrocytes, GFAP (i). Images were taken at the confocal microscope (Zeiss LSM 510 Meta). Scale bar: 10 µm (c and f), 5 µm (c′), 50 µm (d–i) [Color figure can be viewed at http://wileyonlinelibrary.com]

Then, to assess if these reacting cells are indeed able to undergo terminal differentiation at inflammatory sites and generate new myelinating oligodendrocytes, we took advantage of the first transgenic inducible reporter GPR17‐iCreER^T2^xCAG‐eGFP mouse line (Figure 4a). Thanks to the activity of a Cre recombinase, in these mice, upon tamoxifen administration, the cells that are expressing GPR17 at that moment are permanently labeled by the green fluorescent protein GFP (GFP^+^ cells; Figure 4a; Viganò et al., 2016) thus allowing us to trace their destiny throughout animal's life. Since GPR17 is only transiently expressed by OPCs and is no longer present in mature myelinating oligodendrocytes, use of this transgenic mouse line is the only possible means to follow *in vivo* the final destiny of the GPR17^+^ pool of OPCs, even when these cells have already physiologically down‐regulated the receptor.

First, to confirm successful transgene recombination and the identity of recombined cells, we analyzed spinal cord sections in healthy GPR17‐iCreER^T2^xCAG‐eGFP mice. Virtually all the recombinant GFP^+^ cells remained within the oligodendrocyte lineage, as nearly all of them were OLIG2^+^ (Figure 4b,c). Many GFP^+^ cells were also positive for GPR17 (Figure 4d) and NG2 (Figure 4e) suggesting that, 5 weeks after tamoxifen treatment, they were still at a precursor stage. Although they were not abundant, several GFP^+^/GSTπ^+^ cells were also detected (Figure 4f), suggesting that a fraction of the initial GPR17^+^ OPCs indeed underwent maturation. Vice versa, no co‐localization of GFP^+^ cells was found with markers of microglia, neurons, astrocytes (IBA1, NEUN, and GFAP, respectively; Figure 4g–i), as already demonstrated in the brain of these mice (Viganò et al., 2016).

**Figure 5:**
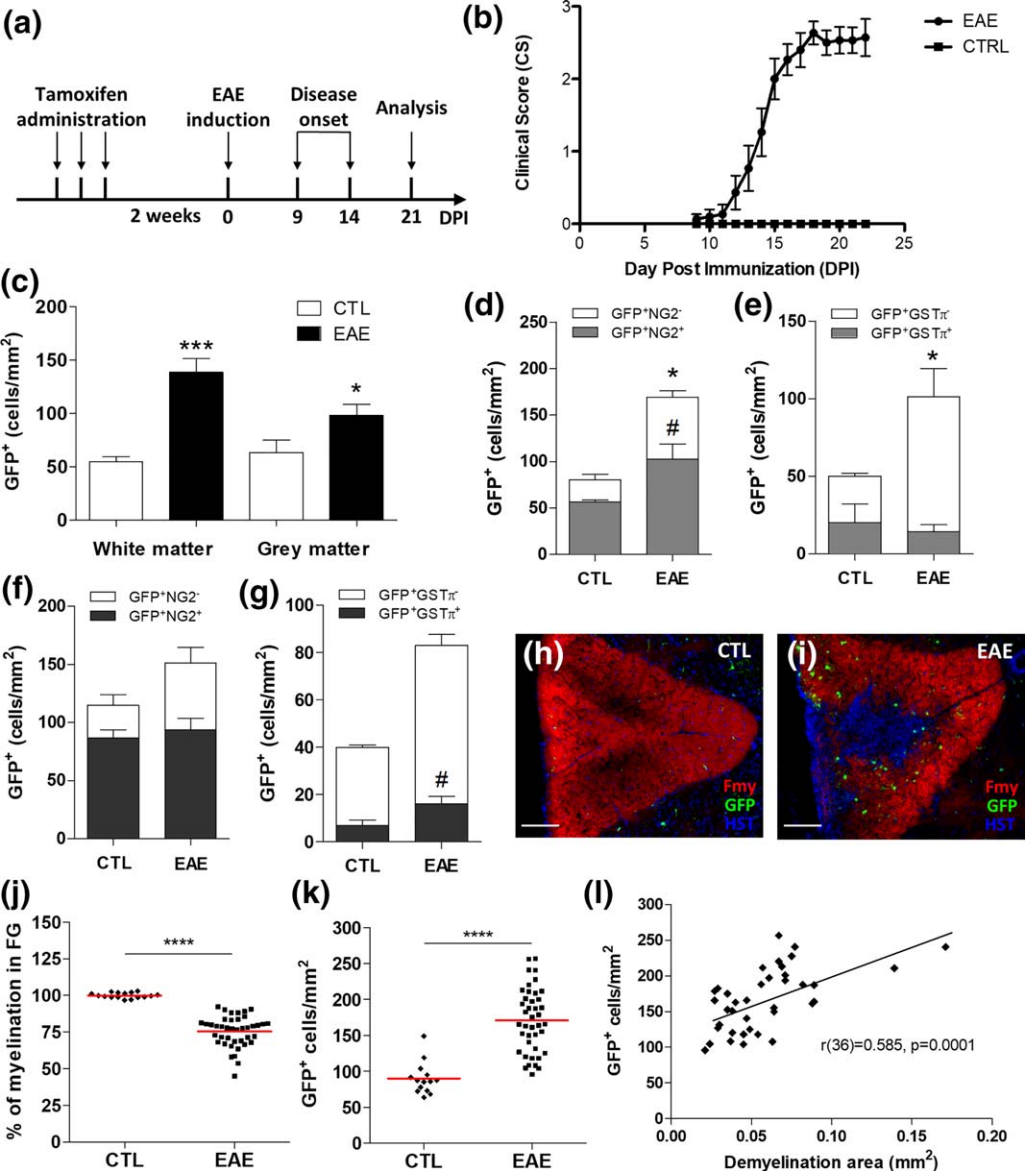
Reaction and fate of recombined cells in the spinal cord of GPR17‐iCreER^T2^xCAG‐eGFP mice after acute EAE. (a) Mice received tamoxifen by oral gavage three times (every other day), starting 14 days before EAE induction, and were analyzed in the EAE acute phase (day 21 after immunization). (b) Clinical scores of mice during acute EAE. Error bars represent mean of CS ± *SEM*. (c) Quantification of the number of GFP^+^ cells in whole spinal cord white (WM) and grey (GM) matter of EAE mice compared with controls. Quantification of GFP^+^/NG2^+^‐ and GFP^+^/GSTπ^+^‐ cells in both WM (d and e) and GM (f and g) in spinal cord of EAE mice. Data are the mean ± *SEM*; (CTL *n* = 2, EAE *n* = 5). Unpaired two‐tailed Student's *t* test; #*p* < .01, *p < .05, ***p < .001 compared with control. (h and i). Representative micrographs showing myelination (fluoromyelin red staining), GFP^+^ cells (green fluorescence) and nuclei (in blue) in the area of the fasciculus gracilis (FG) in control and EAE mice. The accumulation of nuclei in the FG corresponds to infiltrating cells. A decrease in myelination level coupled to a strong increase in the number of GFP^+^ cells was found in the area. Scale bars: 100 µm. The distribution graphs show the percentage of myelination (j) and the number of GFP^+^ cell/mm^2^ (k) in the fasciculus gracilis of the spinal cord in EAE mice compared with controls. Data are the mean ± *SEM* of cervical, thoracic and lumbar sections; (CTL *n* = 15, EAE *n* = 41). Unpaired two‐tailed Student's *t* test; ****p < .0001. (l) The number of GFP^+^ cells was correlated to the extent of demyelination for different FG sections of EAE mice by performing a Pearson's correlation test (*r* = .585; *p* value = .0001). The analysis showed a strong association between the two variables [Color figure can be viewed at http://wileyonlinelibrary.com]

Then, we determined the fate of recombined GFP^+^ cells after EAE using the same protocol described above. To avoid any pharmacological interference with disease onset (Tripathi, Rivers, Young, Jamen, & Richardson, 2010), tamoxifen was administered to adult GPR17‐iCreER^T2^xCAG‐eGFP transgenic mice 2 weeks before EAE induction (protocol in Figure 5a). Analysis of clinical scores confirmed the expected disease development (Figure 5b), suggesting that these mice react to EAE induction as wild‐type animals, and that tamoxifen does not influence EAE susceptibility. Spinal cord sections from these mice were analyzed by immunofluorescence at day 21 after immunization. Quantification of the number of GFP^+^ cells in whole spinal cord sections revealed significant increases in the WM, and, to a lesser extent, in the GM of EAE mice compared with controls (Figure 5c). A more detailed analysis showed that, after EAE, in WM the number of the GFP^+^ cells that also expressed NG2 (i.e., cells that were still at a precursor stage) significantly increased, whereas the number of more mature GFP^+^ cells expressing the more advanced marker GSTπ, did not change (Figure 5d,e). Conversely, in GM, where demyelination was not evident, the number of GFP^+^/NG2^+^ cells did not change, but the number of GFP^+^/GSTπ^+^ cells raised significantly (Figure 5f,g). Globally, these data suggest that the population of GFP^+^ cells is reacting in the WM, mostly by expanding the pool of precursors. However, this expansion is not followed by cell maturation, suggesting that the differentiating abilities of these cells are impaired. Instead, in the GM, where less inflammation and no significant expansion of the GFP^+^ cells were found, a fraction of cells that expressed GPR17 at the time of tamoxifen administration underwent maturation, and acquired the myelin marker GSTπ. We postulate this different behavior of GFP^+^ cells in the GM compared with the WM be due to different extrinsic determinants in these two spinal cord regions, and that, in the WM, despite the local expansion of the NG2^+^ OPC pool, higher demyelination‐associated inflammation contributes to impaired OPC maturation. To corroborate the potential link between GFP^+^ cell response and the extent of demyelination, we focused our subsequent analysis on spinal cord fasciculus gracilis, an area in which most EAE sections invariably show marked demyelination and infiltrating cells (Massella et al., 2012). Representative images showed a marked increase of GFP^+^ cells in section from EAE compared with control mice; these cells were clearly found in the WM close to the central demyelinated area identified by lack of fluoromyelin red staining (Figure 5h,i). We then quantified the demyelination degree in 41 fasciculus gracilis of EAE mice compared with 15 control sections. By setting to 100% the fluoromyelin signal of controls, a mean demyelination degree of 24.6% was detected in EAE mice (Figure 5j) In the same area, the density of GFP^+^ cells was increased to 189.8% (Figure 5k) in the EAE compared with control group set to 100%. Correlation analysis confirmed a strong association between the number of GFP^+^ cells and the extent of demyelination (Figure 5l).

### 3.3 GPR17‐expressing OPCs respond to toxic damage by expanding their pool and contributing to remyelination in the cuprizone model

**Figure 6:**
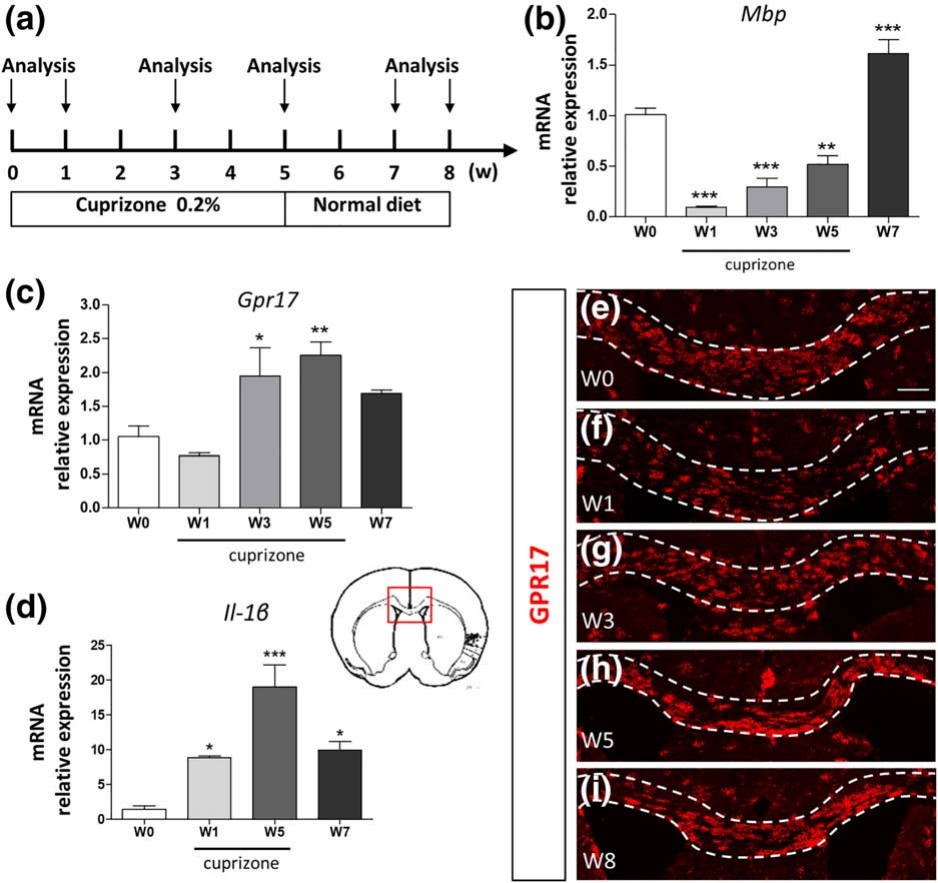
Reaction of GPR17^+^ OPCs in the corpus callosum of cuprizone fed mice. (a) Mice received a 2% cuprizone‐supplemented diet for up to 5 weeks (W) to induce demyelination and were then switched to normal diet to allow spontaneous remyelination. Expression profile of *Mbp* (b), *Gpr17* (c), and *IL‐1β* (d) genes in the corpus callosum during demyelination (1, 3, and 5 weeks of cuprizone diet indicated as W0, W1, W3, and W5) and during the remyelination phase after cuprizone withdrawal (7 weeks, 7W). *Mbp* expression followed the typical already published pattern, with a marked decrease at W1–W5, followed by recovery at W7; in the case of *Gpr17*, an initial decrease was followed by increased expression at later phases of demyelination (3W and 5W). *Gpr17* up‐regulation persisted also after cuprizone withdrawal (W7). As expected, *IL‐1β* expression increased overtime, peaking at W5. Data are the mean ± *SEM* of four animals for each time‐point; one‐way ANOVA with Tukey's multiple comparison post test; *p < .05, **p < .01, ***p < .001 compared with W0. (e–i) Qualitative immunostaining showing GPR17 expression (in red) during demyelination and remyelination phases in corpus callosum (highlighted by the white dotted lines). The red inset in the brain's drawing refers to the area where the IHC analysis reported in e–i was performed. Scale bar = 200 µm [Color figure can be viewed at http://wileyonlinelibrary.com]

In the EAE model, both inflammation and demyelination are very strong due to T‐cell activation and inflammatory infiltrates, and remyelination occurs to a limited extent. Thus, to evaluate the importance of immune activation and inflammation in the changes of GPR17^+^ OPCs, we took advantage of the cuprizone model, a toxic‐induced demyelination paradigm in which immune system is not involved, inflammation is transient, and the kinetics of both demyelination and the subsequent remyelination phase are clearly defined (Supporting Information Figure S1). To this purpose, wild‐type mice received a cuprizone‐supplemented diet for 5 weeks to cause demyelination and were then switched to normal diet for further 3 weeks to allow spontaneous remyelination (Figure 6a). As expected, in corpus callosum expression of the myelin gene *Mbp* showed typical time‐dependent changes consisting in an immediate decrease already evident after 1 week (W1), followed by a rapid recovery after cuprizone withdrawal (Figure 6b). *Gpr17* expression showed a trend to decrease at W1 followed by rapid and significant upregulation peaking at W5 and returning toward control levels after cuprizone withdrawal (Figure 6c), with kinetics similar to those of the proinflammatory cytokine *IL‐1β* (Figure 6d).

The time dependent changes of GPR17 were also confirmed by IHC data. After an initial decrease, due to cuprizone‐induced cell loss (W1; Figure 6e,f), during the demyelination phase we observed a strong accumulation of cells expressing the GPR17 receptor protein in corpus callosum (W3–5; Figure 6g,h), suggesting that OPCs were responding to damage and started to differentiate.

In line with what observed for the *Gpr17* gene (Figure 6c), during the remyelination phase, the number of GPR17^+^ cells in corpus callosum peaked at W5 and then returned to basal levels at W8 (Figure 6i).

**Figure 7:**
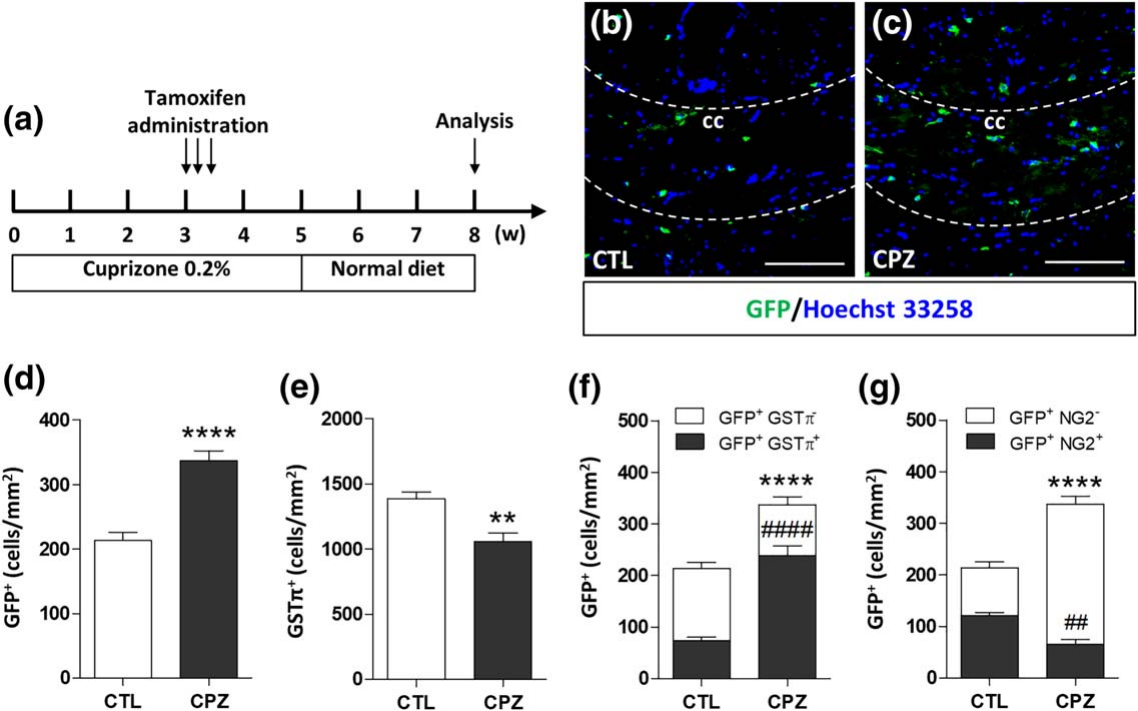
The pool of expanded GPR17^+^ OPCs undergoes terminal maturation in response to cuprizone‐induced demyelination. (a) GPR17‐iCreER^T2^xCAG‐eGFP mice received a 0.2% cuprizone (CPZ)‐supplemented diet for up to 5 weeks (W) to induce demyelination and were then switched to normal diet to allow spontaneous remyelination. Tamoxifen induction was performed at 3W after beginning the cuprizone diet. Analyses were performed at the end of the experimental protocol (8W). (b and c) Qualitative immunostaining showing GFP expression (in green) in corpus callosum (cc) merged with Hoechst 33258 (in blue) to label cell nuclei. (d and e) Quantification of the number of GFP^+^‐cells and GSTπ^+^ cells in cc of CPZ fed mice compared with mice receiving standard diet (CTL). (f and g) Quantification of the number of GFP^+^/GSTπ^+^ cells and GFP^+^/NG2^+^ compared with GFP^+^ cells in cc of CPZ fed mice (*n* = 3). Data are the mean ± *SEM*. Unpaired *t* test; **p < .01, ****p < .0001, ####*p* < .0001, compared with mice receiving the standard diet (CTL). Scale bar = 25 µm [Color figure can be viewed at http://wileyonlinelibrary.com]

Then, to assess whether, in this demyelinating model, GPR17^+^ reacting cells undergo differentiation or stay blocked at immature stages as in the EAE model, we fed GPR17‐iCreER^T2^xCAG‐eGFP mice with cuprizone, and, at W3, induced GFP expression with tamoxifen (Figure 7a). This specific timing of tamoxifen administration was chosen to properly monitor the final fate of the OPCs that were expressing GPR17 at the beginning of the OPC proliferation wave (Gudi et al., 2014). At W8, mice were sacrificed and the maturation stage of the reacting GPR17^+^ cells was assessed by double labeling for both GFP and, either NG2 (a marker of oligodendrocyte precursors) or GSTπ (a marker of mature oligodendrocytes). Similarly to EAE, a marked increase in the number of GFP^+^ cells (Figure 7b–d) was found in the corpus callosum of cuprizone fed mice compared with controls, suggesting a damage‐induced expansion of the GPR17^+^ pool of cells at W3. In this model, a role for OPCs originating from the subventricular zone (SVZ) has been also suggested (Xing et al., 2014). However, our staining showed a very low induction of GPR17 in this area (data not shown), suggesting a minor involvement of SVZ‐derived GFP^+^ cells in the remyelination process.

Although the number of GSTπ^+^ cells was still lower in the corpus callosum of cuprizone fed mice compared with control (Figure 7e), in the cuprizone group, a significantly higher number of OPCs stained for both GSTπ and GFP was found (Figure 7f), suggesting that many of the OPCs expressing GPR17 at W3 had been able to reach terminal maturation after cuprizone withdrawal. Accordingly, at W8, the number of early GFP^+^ precursors still expressing NG2 was lower in the cuprizone group compared with controls (Figure 7g).

Globally, these data suggest that, at variance from EAE, in this model of demyelination, GPR17^+^ OPCs are able to react to the toxic damage induced by cuprizone administration by first expanding their pool and then progressing to terminally differentiated cells, thus effectively participating to remyelination.

## 4 DISCUSSION

In recent years, GPR17 has emerged as an important key actor in oligodendrogenesis. Previous studies have shown that, in the brain, GPR17 acts as an intrinsic regulator of this process: indeed, it is necessary to start OPC differentiation but, after a certain stage, it has to be turned down to allow oligodendrocyte terminal maturation (Fumagalli et al., 2015; Fumagalli, Lecca, Coppolino, Parravicini, & Abbracchio, 2017). These features have led to the proposal that GPR17 represents a new potential target for remyelination therapies in diseases characterized by myelin disruption such as multiple sclerosis. Here, for the first time, we define the differentiation capabilities of GPR17^+^ cells in rodents after myelin disruption in two different models, the former characterized by diffused immune response against myelin components and strong inflammation (the EAE model), and the latter one consisting in local demyelination induced by a toxic agent (the cuprizone model).

In physiological conditions, only a subset of OPCs (typically 30%–40%) express GPR17 (Boda et al., 2011; Viganò et al., 2016) in both brain and spinal cord, suggesting a role in the normal homeostasis and in oligodendrocyte turnover. Here, we confirm that GPR17^+^ cells are widespread throughout the whole spinal cord, both in the GM and WM, showing co‐localization with both the early marker NG2 and the more advanced marker CC1.

As expected, in the EAE model, 21‐days after immunization, there was a general depletion of oligodendrocytes, as shown by a trend to decrease in OLIG2^+^ and GPR17^+^ cells (Figure 2a) and a statistically significant reduction of mature CC1^+^ cells (Figure 2b), suggesting that mature oligodendrocytes are likely to be more affected by disease induction. At this stage, the total number of spinal cord NG2^+^ cells was unaltered in both the GM and WM (Figure 2c). However, double immunofluorescence analysis on these precursors showed that the NG2^+^/GPR17^+^ subpopulation was increased in the WM but not in the GM, which was indeed relatively spared (Figure 3a), demonstrating that, (i) the GPR17^+^/NG2^+^ subset of cells reacts to the insult, and that (ii) this behavior is restricted to the region where demyelination occurs.

Detailed fate mapping analysis in inducible GPR17‐iCreER^T2^xCAG‐eGFP reporter mice, that allows monitoring the behavior and destiny of GPR17^+^ cells by tamoxifen‐induced GFP labeling, confirmed a strong increase in the number of GFP^+^ cells in damaged areas in both demyelinating models. These results are in line with previous literature data showing that, after injury, OPCs enhance their pool of progenitors, are actively recruited at the sites of focal CNS injury, and rapidly migrate and proliferate to restore their density (Hughes, Kang, Fukaya, & Bergles, 2013). However, we also show that only in the cuprizone model GFP^+^ cells do actually differentiate to mature oligodendrocytes contributing to remyelination (Figure 7f). In EAE spinal cord, significant maturation of GPR17^+^ cells was only observed in the GM (Figure 5g), which is minimally affected by damage. This is in contrast with literature data showing that, in general, OPCs maturate more rapidly in the WM compared with GM (Viganò, Mobius, Gotz, & Dimou, 2013; Viganò et al., 2016), suggesting that, in EAE, specific local extrinsic factors are responsible for altered OPC behavior. We indeed postulate that, in the EAE model, the inability of WM GFP^+^ cells to maturate can be due, at least in part, to the presence of a strong chronic proinflammatory environment blocking cells at immature stages and preventing their terminal maturation. To further support this hypothesis, cuprizone‐induced demyelination mainly evolves as a toxic insult with much less involvement of immune cells leading to less inflammation, likely resulting in a more permissive local environment for OPC maturation (Gudi et al., 2014).

Of course, we cannot exclude that such differential responses can also be due, at least in part, to different OPCs intrinsic programs in spinal cord and corpus callosum. Adult OPCs indeed originate from evolutionary distinct populations, and for this reason they keep different physiological features and capabilities to respond to demyelination for their whole life (Ornelas et al., 2016).

Consistent with our data, the role of GPR17 in addressing OPCs to maturation has been recently confirmed. Indeed, in line with the already postulated heterogeneity of oligodendroglial precursors (Viganò & Dimou 2016) a recent transcriptome analysis identified 12 distinct populations as a continuum in the differentiation process suggesting that the term “OPCs” represents a strong approximation of the actual genic and functional diversity of these cells. Of interest, GPR17 was identified in three of these populations defined as “differentiation committed precursors” (Marques et al., 2016). This confirms that our GPR17 reporter mouse line is a useful tool to fate‐map a specific subpopulation of intermediate precursors committed to maturation, rather than simply highlighting a general “prototypic” early response of NG2‐cells.

Interestingly, the lack of OPC terminal maturation in EAE tissues is also accompanied by persistent GPR17 overexpression. As already mentioned, while GPR17 is needed to start differentiation, its chronic overexpression due to impaired physiological downregulation has been associated to OPC blockade and impaired remyelination (Fumagalli et al., 2015; Ou et al., 2016). Besides EAE (the present study), several other distinct models of brain disease, including stroke, trauma, Alzheimer's (Fumagalli et al., 2016) have been associated to persistent GPR17 upregulation and impaired progression along the oligodendrocyte lineage, eventually resulting in dysfunctional repair. Importantly, GPR17 has been shown to “promiscuously” respond to several different proinflammatory ligands accumulating at injury sites (Sensi et al., 2014; Parravicini et al., 2016), not only uracil nucleotides and cysteinyl‐leukotrienes (Ciana et al., 2006), but also oxysterols and CXCL12 (Parravicini et al., 2016), one of the most prominent chemokines in the lesions of multiple sclerosis patients (Calderon et al., 2006). In a permissive environment, these inflammatory stimuli could initially contribute to promotion of remyelination through GPR17 stimulation, in line with literature data proposing that acute inflammation triggers remyelination (Foote & Blakemore 2005). However, when inflammation becomes stronger and chronic (as it occurs when EAE is overtly symptomatic), this beneficial effect is turned into a detrimental condition (Marchetti & Abbracchio 2005) that worsens rather than favor, remyelination. In line with this hypothesis, the strong inflammatory milieu associated to the degenerative conditions mentioned above is accompanied by GPR17 upregulation (Fumagalli et al., 2015), which prevents terminal OPC maturation and remyelination.

Based on the above evidence, on the strikingly different contribution of inflammation to EAE‐ and cuprizone‐induced demyelination (Denic et al., 2011; Gudi et al., 2014), and based on the different capability of GPR17^+^ OPCs to undergo maturation in these two rodent models (the present results), we speculate that the proinflammatory local environment associated to EAE induction is responsible for persistent GPR17 overexpression in OPCs, leading to their blockade at immature stages.

Our data open new directions to treat demyelination in neuroinflammatory diseases, such as multiple sclerosis, in which inappropriate resolution of acute inflammation leads to its chronicization. They also suggest that remyelinating approaches should be combined to therapies controlling chronic inflammation, in order to achieve effective myelin repair and retard neurodegeneration.

## CONFLICT OF INTEREST

The authors declare that they have no conflict of interest.

## Supporting information

Additional Supporting Information may be found online in the supporting information tab for this article.

Supporting Information Figure_S1
