# Supplementary figures and images for: Differential local tissue permissiveness influences the final fate of GPR17‐expressing oligodendrocyte precursors in two distinct models of demyelination
Source: Glia. 2018 Feb 9;66(5):1118–30. doi: 10.1002/glia.23305 (PMC5900886; doi:10.1002/glia.23305)

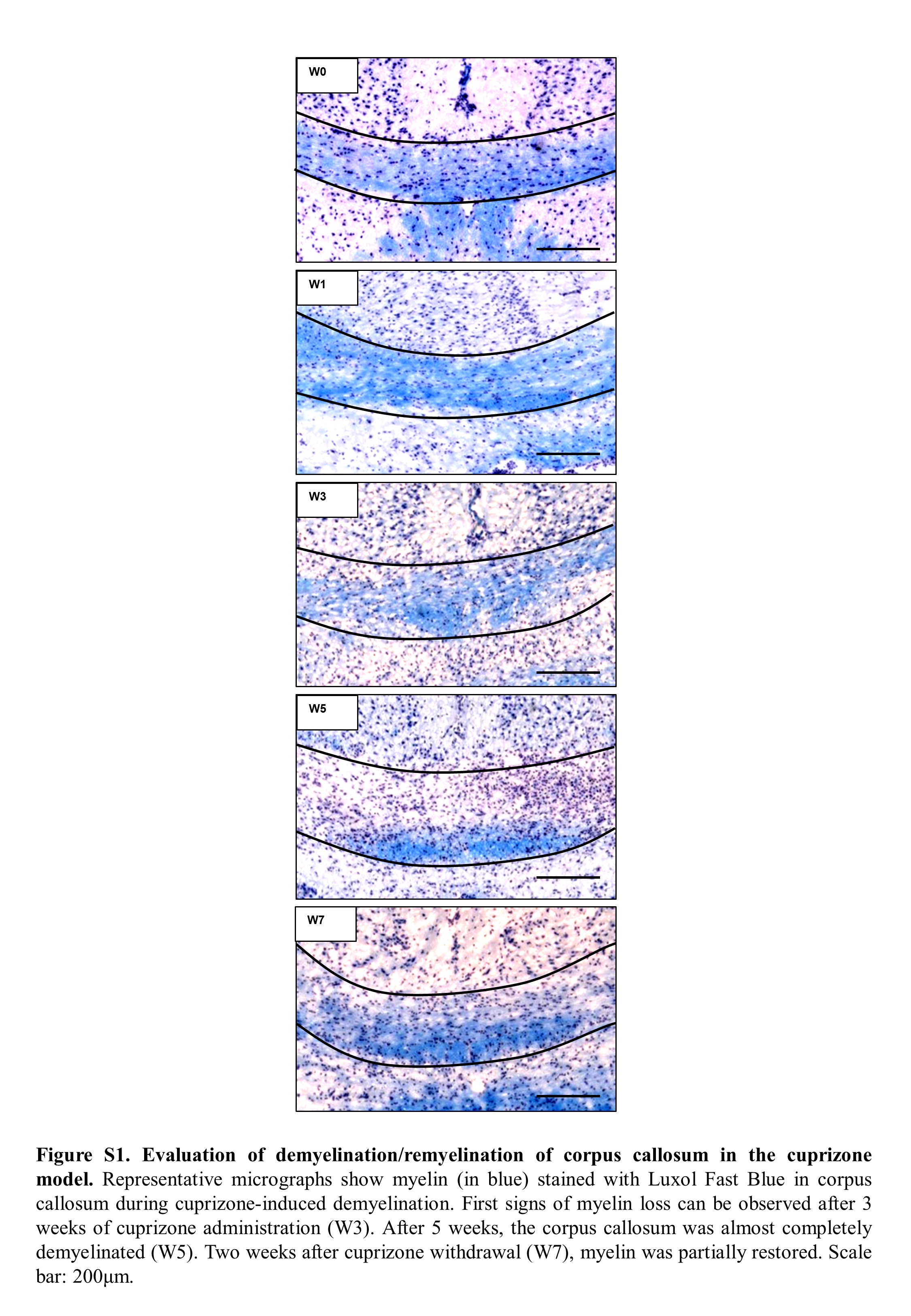

Supplement: Supplementary file 1 — Supporting Information Figure_S1 [file GLIA-66-1118-s001.tif]
